# Effect of time factors on the mortality in brittle hip fracture

**DOI:** 10.1186/1749-799X-9-37

**Published:** 2014-05-16

**Authors:** Yizhong Li, Jinkuang Lin, Peiwen Wang, Xuedong Yao, Haiming Yu, Huafeng Zhuang, Linlin Zhang, Yanjun Zeng

**Affiliations:** 1 Department of Orthopedics, the Second Affiliated Hospital of Fujian Medical University, Quanzhou City, Fujian Province 362000, China; 2 Beijing University of Technology, Beijing 100022, China

**Keywords:** Time factors, Hip fracture, Mortality

## Abstract

**Objective:**

The aim of this research is to study the effect of time factors on the mortality of brittle hip fracture.

**Methods:**

The data of 705 patients of hip fracture hospitalized at our department from 2010 to 2012 were reviewed. Among them, 499 cases undergoing surgical operation over the age of 50 were followed up, and 250 cases had valid follow-up records. The effects of the time from injury to operation, the time from injury to hospitalization and the time from hospitalization to operation on the mortality were analyzed.

**Results:**

The average duration of follow-up was 21.37 ± 9.77 months. There were 198 cases which were followed up for over 12 months. Nine patients died within 3 months after the hip fracture surgery, and 13 patients died within 1 year. A total of 25 patients died during the follow-up. The survival rate of patients with the interval from injury to surgery longer than 5 days was lower than that of patients with the interval less than or equal to 5 days (*p* = 0.014). The survival rate of patients with the interval from injury to hospitalization longer than 2 days was lower than that of patients with the interval from injury to hospitalization less than or equal to 2 days (*p* = 0.003). There was no statistical significance in the survival rate between patients with the interval from hospitalization to surgery longer than 3 days and that of patients with the interval from hospitalization to surgery less than or equal to 3 days (*p* = 0.973).

**Conclusion:**

The operational delay, especially the delay of time from injury to hospitalization, is an important factor affecting the early mortality of hip fracture. The delay of time from hospitalization to operation is mainly due to the consideration of the patients' situation and has no effect on early mortality.

## Introduction

Hip fracture is a severe osteoporotic fracture. The incidence rate among males over the age of 50 in China is 129/100 thousand, and that of female is 229/100 thousand. There are about 1.6 million patients suffering from hip fracture around the world each year. It is expected that the hip fracture events will increase to 4.5–6.3 million by 2050 [1,2]. The deaths from complications associated with hip fracture within 1 year reach 20%–30%. The disability rate is up to 50%. The effect of hip fracture on death can extend over 10 years [3-6].

The effect of operational delay on mortality has always been the focus of debate. It is believed in many studies that the operational delay will lead to the rise of 1-year mortality. But other studies find that operational delay has no correlation with 1-year mortality. These studies are mainly about the effect of the delay from hospitalization to surgery on the mortality. Different conclusions obtained are possibly correlated with the level of medical care of hospital and the definition of operational delay. Therefore, it is necessary to distinguish between the reasons of operational delay: operational delay for the stabilization of the patients' situation or unnecessary operational delay. The effect of time interval from injury to surgery on the mortality of hip brittle fracture should be observed. The interval from injury to surgery was also divided into two time periods, namely, from injury to hospitalization and from hospitalization to surgery. It can be accurately known which of the two time factors affects the mortality.

The medical knowledge possessed by patients and their relatives affects the length of time from injury to hospitalization. The delay in this period contributes very little to the preoperative preparation. The level of medical care of hospital determines the time from hospitalization to operation. Such operational delay may be indispensable for preoperative preparation and the stabilization of the patients' condition. Both are the operational delay. The understanding on the influence of the two time factors on the mortality of hip fracture is beneficial for formulating more effective countermeasures. In order to investigate the effect of time factor on the mortality of hip fracture, the data of patients with hip fracture from January 2010 to December 2012 were studied retrospectively.

## Data and methods

This research was carried out in the Second Affiliated Hospital of Fujian Medical University. It was permitted by both the hospital and the patients. The research was carried out strictly according to the requirements of documents issued by China's Ministry of Science and Technology and Ministry of Public-Health. Relevant obligations and responsibilities with respect to requirements in human ethics were undertaken by the research group.

The medical data of 705 patients with hip fractures hospitalized from January 2010 to December 2012 were retrospectively studied. Excluding the patients with pathological fractures caused by malignant tumors, those aged below 50 years old and those with hip fractures caused by high energy violence and not treated by operation, there were finally 499 cases with brittle hip fractures aged ≥50 and undergoing operation treatment (70.78%). The patients receiving the surgery for hip brittle fracture from January 2010 to December 2012 were followed up, more than 6 months after the surgery. In this follow-up visit, the physicians visited the patients or the family members who lived with the patients before death, and the valid records were obtained from 250 cases (50.10%). The patients' medical records admitted with hip fracture surgery were retrospectively reviewed. Among 250 patients aged from 50 to 97, the average age was 76.70 ± 10.77. There were 83 males with the average age of 73.64 ± 11.40, and 167 females with the average age of 78.21 ± 10.16. The female-to-male ratio was 2.01. There were 127 cases with fractures of femoral neck, including 33 males and 94 females. The average age was 75.36 ± 10.67. There were 123 cases with intertrochanteric fractures, including 50 males and 73 females. The average age was 78.40 ± 10.70. There were 185 cases combined with internal diseases, accounting for 74%; 67 cases were combined with one type of internal disease (26.8%), 85 with two types of internal disease (34%), and 33 with more than or equal to three types of internal disease (13.2%). The main combined diseases included cardiovascular and cerebrovascular diseases, diabetes mellitus, chronic respiratory disease, anemia, hypoproteinemia, liver cirrhosis, and renal insufficiency.

### Observed indicators

The time periods from injury to hospitalization, from hospitalization to operation, and from injury to operation were set up respectively in order to observe the effects of the above three time factors on the mortalities.

1. Time from injury to operation: The time from injury to operation can well represent whether the operation is delayed. Therefore, it was more meaningful to particularly observe its effect on mortality. The patients were divided into ≤5-day group and >5-day group to observe the effects of time on mortality.

2. Time from injury to hospitalization: The patients were divided into two groups, ≤2-day group and >2-day group for the time from injury to hospitalization to observe the effects of different lengths of time from injury to hospitalization on mortality.

3. Time from hospitalization to operation: The patients were divided into ≤3-day group and >3-day group for the time from hospitalization to operation to observe the effects of different lengths of time from hospitalization to operation on mortality.

## Statistical method

All statistical analyses were performed using the SPSS16.0 software. The measurement data were expressed as mean ± standard deviation. The intergroup comparison was performed by using the chi-square test. The comparison of survival rate was made by the Cox proportional hazards analysis. It was considered that there was a statistically significant difference when *p* < 0.05.

## Results

According to the grouping method based on the time from injury to operation, from injury to hospitalization, and from hospitalization to operation, the clinical data of patients in each group were combined (Tables 1, 2, 3). The incidence rates of complications in patients with time >5 days from injury to operation were significantly higher (*p* < 0.05) than those of the patients with time ≤5 days. The sites of hip fracture were different between the patients with the time from injury to hospitalization of ≤2 days. The incidence rates of complications (combined with two types of complications) in the patients with the time from hospitalization to operation of >3 days and pulmonary infection rates were significantly higher (*p* < 0.05) than those of the patients with time of ≤3 days.

**Table 1:** Data of patients with the time from injury to operation of ≤5 and >5 days

	**≤5 days**	**>5 days**	***p***
Cases	127	123	
Age (years)	75.0 ± 11.5	78.5 ± 9.7	0.010
Male	42 (33.1%)	41 (33.3%)	0.929
Female	85 (66.9%)	82 (66.7%)	0.929
Fracture of femoral neck	58 (45.7%)	69 (56.1%)	0.099
Intertrochanteric fracture	69 (54.3%)	54 (43.9%)	0.099
Complications	81 (63.8%)	100 (81.3%)	0.002
Combined with one internal disease	43 (33.9%)	24 (19.5%)	0.010
Combined with two types of internal disease	31 (24.4%)	54 (43.9%)	0.001
Combined with more than or equal to three internal diseases	7 (5.5%)	22 (17.9%)	0.002
Pulmonary infection	18 (14.2%)	27 (22.0%)	0.110

**Table 2:** Data of patients with the time from injury to hospitalization of ≤2 and >2 days

	**≤2 days**	**>2 days**	***p***
Cases	182	68	
Age (years)	76.0 ± 11.2	78.6 ± 9.5	0.080
Male	59 (32.4%)	24 (35.3%)	0.667
Female	123 (67.6%)	44 (64.7%)	0.667
Fracture of femoral neck	81 (44.5%)	46 (67.6%)	0.001
Intertrochanteric fracture	101 (55.5%)	22 (32.4%)	0.001
Combined internal disease	136 (74.7%)	50 (73.5%)	0.847
Combined with one type of internal disease	55 (30.2%)	13 (19.1%)	0.079
Combined with two types of internal disease	57 (31.3%)	27 (39.7%)	0.212
Combined with ≥3 internal disease	24 (13.2%)	10 (14.7%)	0.755
Pulmonary infection	32 (17.6%)	13 (19.1%)	0.793

**Table 3:** Data of patients with the time from hospitalization to operation of ≤3 days and >3 days

	**≤3 days**	**>3 days**	***p***
Cases	96	154	
Age (years)	75.6 ± 11.4	77.3 ± 10.4	0.227
Male	34 (35.4%)	49 (31.8%)	0.557
Female	62 (64.6%)	105 (68.2%)	0.557
Fracture of femoral neck	49 (51.0%)	78 (50.6%)	0.952
Intertrochanteric fracture	47 (49.0%)	76 (49.4%)	0.952
Combined with internal disease	60 (62.5%)	124 (80.5%)	0.015
Combined with one type of internal disease	25 (26.0%)	43 (27.9%)	0.745
Combined with two types of internal disease	24 (25.0%)	60 (39.0%)	0.023
Combined with more than or equal to three internal diseases	11 (11.5%)	21 (13.6%)	0.616
Pulmonary infection	11 (11.5%)	33 (21.4%)	0.044

**Figure 1:**
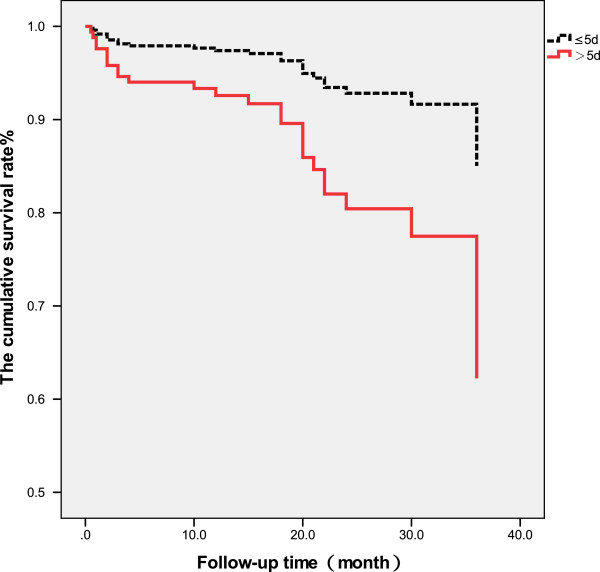
Survival rate of patients with interval from injury to surgery of >5 and ≤5 days.

**Figure 2:**
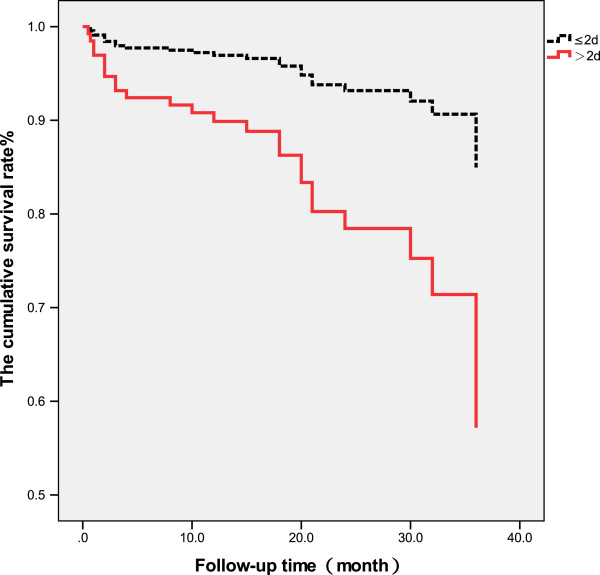
Survival rate of patients with interval from injury to hospitalization >2 and ≤2 days.

**Figure 3:**
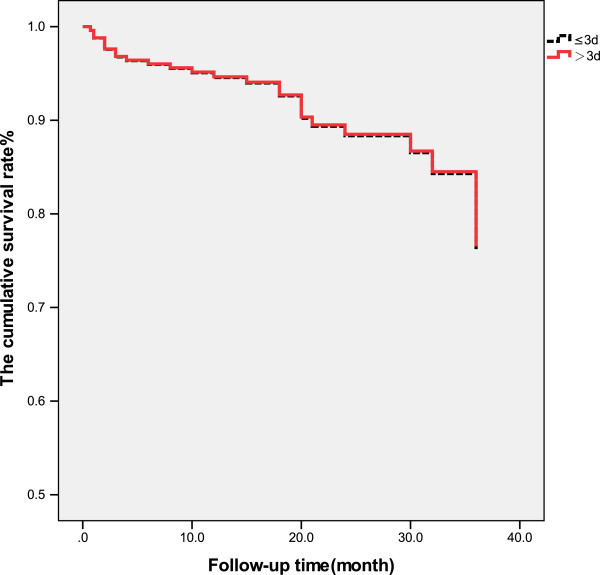
Survival rate of patients with interval from hospitalization to surgery >3 and ≤3 days.

The patients were followed up to 42 months after receiving surgery (mean 21.37 ± 9.77 months) when 9 patients had died after 3 months, and 13 patients had died within 1 year after surgery. A total of 25 patients died during the follow-up. The survival rate of patients with interval from injury to surgery longer than 5 days was significantly lower than that of patients with interval less than or equal to 5 days (*p* < 0.05, Figure 1). The survival rate of patients with interval from injury to hospitalization longer than 2 days was significantly lower than that of patients with the interval less than or equal to 2 days (*p* < 0.01, Figure 2). There was no significant difference in the survival rate between patients with interval from hospitalization to surgery longer than 3 days than that of patients with the interval less than or equal to 3 days (*p* > 0.05, Table 4, Figure 3).

**Table 4:** Statistical result of survival rate

**Time factors**	**Number of cases**	**Survival rate (%)**	**HR**	**95% confidence interval**	***p *** **value**
A					
≤5 days	127	94.49	0.34	0.15–0.81	0.014
>5 days	123	85.37			
B					
≤2 days	182	93.41	0.29	0.13–0.65	0.003
>2 days	68	80.88			
C					
≤3 days	96	88.54	1.01	0.46–2.25	0.973
>3 days	154	90.91			

*A* time from injury to surgery, *B* time from injury to hospitalization, *C* time from hospitalization to surgery.

## Discussion

Most of the patients with hip fracture are combined with internal disease, and surgical operation is the topic choice of treatment [7,8]. The combination of internal diseases is often an important factor affecting the decisions made by physicians on the time of surgical treatment for hip fracture. In our study, 74.2% of the patients were combined with internal diseases, and 47.2% with two or more than two internal diseases. This was an important reason for the delay of operation. There are many risk factors for the death of patients with brittle hip fracture. However, the effect of the delay of surgical operation on mortality is the most controversial. Through the systematic review and meta-analysis by Simunovic et al. [9] of 16 studies, it was considered that the mortality, postoperative pneumonia, and bedsore can be decreased by early operation (<24, <48, or <72 h). It was reported by Novack et al. [10] that the patients undergoing the operation within 48 h after hip fracture had the lowest mortalities during the hospitalization period as well as the lowest 1-month and 1-year mortalities. The early mortalities and 1-year mortalities would rise when the operation was performed after 48 h. Early operation can lower the time in bed, thereby reducing the postoperative risks for patients with brittle hip fracture, including serious infections, venous thrombosis, and death [11,12]. Decreasing the operational delay is the most important factor to reduce the mortalities of patients with hip fracture [13].

However, when early operation cannot be performed or other medical needs cannot be met, there should be sufficient time for the treatment of the elderly patients combined with internal diseases before operation. Preoperative preparation and evaluation should be improved in order to reduce the operation risks and to improve the success rate. Kim et al. [14] reported that the operational delay after hospitalization did not affect the incidence of postoperative complications. It was considered by Vidal et al. [15,16] that there were no correlations between the length of time from hospitalization to operation and mortality during hospitalization period and 1-year mortality after the operation for patients with brittle hip fracture. However, the length of time from injury to hospitalization is the factor affecting the mortality. For the delay of every 1 day from fracture to hospitalization, the mortality risk increased by 9% during the hospitalization, and the 1-year mortality after operation increased by 7%. There were about 10% of patients with hip fracture that were hospitalized only after more than 2 days. Orosz et al. [17] indicated that the main reason for the delay of hospitalization is that the relatives of the patients are not aware of the severity of the injury. The patients were not hospitalized until the bedsore appeared after lying in bed for several days, with no pain alleviation and the combination of pulmonary infection and other diseases. This often results in the delay of treatment and a higher mortality.

In this study, the time interval from injury to surgery was divided into two periods, i.e., time from injury to hospitalization and time from hospitalization to surgery. We expected to discover which time factor affected the mortality of patients with hip fracture. The effects of the length of these two time periods on mortality were observed to understand the real time factor causing the increase of mortality due to operational delay and whether the delay of time from hospitalization to operation is really based on the needs of patients. There were 27.2% of patients with hip fracture that were hospitalized after more than 2 days in our series. It was found that there was an obvious reduction in the statistical significance in the survival rates of patients with the interval from injury to hospitalization exceeding 2 days and those receiving surgery more than 5 days after the injury. The time from hospitalization to surgery did not affect the survival rate of patients. Therefore, the physicians in the community and the workers from public health system should fully understand that the operational delay can increase the death risk of the patients with hip fracture. The most effective measure to avoid the operational delay was the immediate hospitalization of patients.

## Conclusion

It was indicated that the operational delay did increase the mortality of patients with hip fracture. However, the key delay causing the increase of mortality was the delay from injury to hospitalization, and not the delay from hospitalization to operation. The basic-level medical institutions and patients and their relatives must recognize the seriousness of hip fracture and should promptly send the patients to qualified hospitals to reduce the delay of operation. However, the full preparation before the operation is a must to ensure the medical safety.

## Competing interests

The authors declare that they have no competing interests.

## Authors’ contributions

YL, JL, and YZ designed the research and participated in the sequence alignment. PW, XY, and HY performed the statistical analysis. HZ, LZ, and YZ participated in the editing and submission of the paper. All authors read and approved the final manuscript.
